# Mediterranean Diet and NAFLD: What We Know and Questions That Still Need to Be Answered

**DOI:** 10.3390/nu11122971

**Published:** 2019-12-05

**Authors:** Maria Corina Plaz Torres, Alessio Aghemo, Ana Lleo, Giorgia Bodini, Manuele Furnari, Elisa Marabotto, Luca Miele, Edoardo G. Giannini

**Affiliations:** 1 Cattedra di Gastroenterologia, Dipartimento di Medicina Interna, Università degli Studi di Genova, 16132 Genova, Italy; 2 Programma Dipartimentale Diagnosi e Terapia delle Malattie Emergenti dell’Apparato Digerente, IRCCS Ospedale Policlinico San Martino, 16132 Genova, Italy; 3 Divisione di Medicine Interna ed Epatologia, Humanitas Clinical and Research Center-IRCCS, 20089 Rozzano, Italy; 4 Dipartimento di Scienze Biomediche, Humanitas University, 20090 Pieve Emanuele, Italy; 5 Area Medicina Interna, Gastroenterologia e Medicina Interna, Fondazione Policlinico Gemelli IRCCS, Università Cattolica del Sacro Cuore, 20123 Roma, Italy; 6 Gastroenterology Unit, Department of Internal Medicine, University of Genoa, Viale Benedetto XV, no.6, 16132 Genoa, Italy

**Keywords:** nonalcoholic steatohepatitis, chronic liver disease, lifestyle intervention, diet, outcome

## Abstract

Non-alcoholic fatty liver disease (NAFLD) is the hepatic manifestation of metabolic syndrome and is expected to become the leading cause of end-stage liver disease worldwide over the next few decades. In fact, NAFLD encompasses different clinical scenarios, from the simple accumulation of fat (steatosis) to steatohepatitis (NASH), NASH-cirrhosis, and cirrhosis complications. In this context, it is fundamental to pursue strategies aimed at both preventing the disease and reducing the progression of liver fibrosis once liver damage is already initiated. As of today, no pharmacological treatment has been approved for NAFLD/NASH, and the only recommended treatment of proven efficacy are life-style modifications, including diet and physical exercise pointing at weight loss of 5%–7%. Different dietetic approaches have been proposed in this setting, and in this review, we will discuss the evidence regarding the efficacy of the Mediterranean Diet as a treatment for NAFLD. In particular, we will report the effects on liver-related outcomes.

## 1. Introduction

NAFLD is considered the hepatic manifestation of metabolic syndrome (MetS) and is defined as the accumulation of fat in the liver in patients who do not consume excessive amounts of alcohol (i.e., more than 20 g/day for women and less than 30 g/day for men). NAFLD encompasses different clinical scenarios, from the simple accumulation of fat in the liver (steatosis), to steatohepatitis (NASH), cirrhosis, and its complications [1].

The burden of disease related to NAFLD is extremely high in the general population, and the global prevalence of liver steatosis is indeed around 25%, reaching a pike of 46% in the United States [2]. These percentages have gradually increased in the past decades, parallel to rising rates of obesity and diabetes, to which NAFLD is tightly related. It needs to be pointed out that, owing to the increasing prevalence of the disease and to the prolonged life-expectancy, NASH-related cirrhosis is estimated to become the leading cause of end-stage liver disease and liver transplantation in the near future [3,4]. As a fact, NASH-cirrhosis has become one of the most frequent causes of chronic liver disease in developed countries, and is at present one of the leading indications for liver transplantation for both end-stage liver disease (ESLD) and hepatocellular carcinoma (HCC) [5,6]. Moreover, it is widely recognized that NAFLD not only increases liver-related morbidity and mortality, but is also closely related to increased overall mortality and risk of type 2 diabetes, chronic kidney disease, and cardio-vascular diseases [7,8,9]. In this context, the relevance of preventing NAFLD and its eventual complications at the population level can be easily appreciated.

As of today, there are no approved drugs for the treatment of NAFLD, therefore no specific treatment can be recommended, even though the last NAFLD-European Association for the Study of the Liver guidelines suggested that vitamin E could be used as a potential treatment for non-cirrhotic NASH patients as it reportedly improves NASH histological features, although the long-term benefits of vitamin E administration are unknown and must be weighed against the potential occurrence of well-characterized side effects [3]. In clinical practice, the most recommended treatment for NAFLD are lifestyle interventions targeting weight reduction, and such interventions mainly include diet and physical exercise [3].

A variety of dietary patterns have been proposed for NAFLD-treatment but no pattern has adequately been evaluated in randomized controlled trials in large cohorts of patients. However, the Mediterranean diet (MD) may reasonably be an effective and easily reproducible diet pattern, as it is characterized by a balanced composition of macronutrients which have positive effects on NAFLD that can be found in foods from different areas in the world [10,11,12]. The MD has indeed been recently recommended as the diet of choice for the treatment of NAFLD by the EASL-EASD-EASO Clinical Practice Guidelines as it improves metabolism, in particular through a reduction of insulin-resistance and lipid serum concentrations, and it induces a regression of steatosis and a significant reduction of cardio-vascular events [3,13,14,15,16,17].

In the last decade, several studies have been conducted to assess the benefits of MD in NAFLD patients. In this review we will report the current evidence for the effects of MD in this setting, with particular attention to liver outcomes, since its beneficial effects on metabolic syndrome (MetS) have already been extensively described [13,14,15,16].

## 2. Mediterranean Diet Characteristics and Its Potential Effects on NAFLD

The pathogenesis of NAFLD is multifactorial, being related to a complex interaction between environmental factors and genetic predisposition [17]. Insulin-resistance plays a major role in the development of NAFLD and its progression towards NASH through (a) increased hepatic de-novo lipogenesis, (b) impaired inhibition of lipolysis in the adipose tissue with consequent increased flux of fatty acids to the liver, and (c) adipose tissue dysfunction with altered production and secretion of adipokines and inflammatory cytokines [18,19]. The high concentrations of free fatty acids, free cholesterol, and lipid metabolites induce lipotoxicity with the production of reactive oxygen species, consequent mitochondrial dysfunction, and endoplasmic reticulum stress [20]. Another potentially relevant factor involved in the pathogenesis of NAFLD is represented by gut microbiota, which have been shown to be distinct in NAFLD, reportedly leading to increased small bowel permeability, free fatty acids absorption, and the activation and release of pro-inflammatory cytokines [21]. Lastly, a genetic predisposition may be involved in NAFLD pathogenesis [22,23].

**Figure 1:**
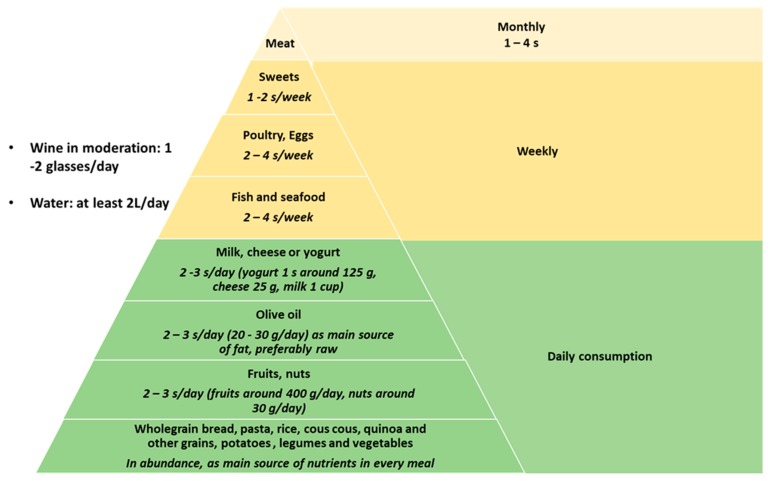
Mediterranean diet pyramid: this figure emphasizes the hierarchical composition of the Mediterranean diet, that is mainly based on the daily consumption of vegetables, fibers, nuts, fruits, dietary products, and olive oil.

**Figure 2:**
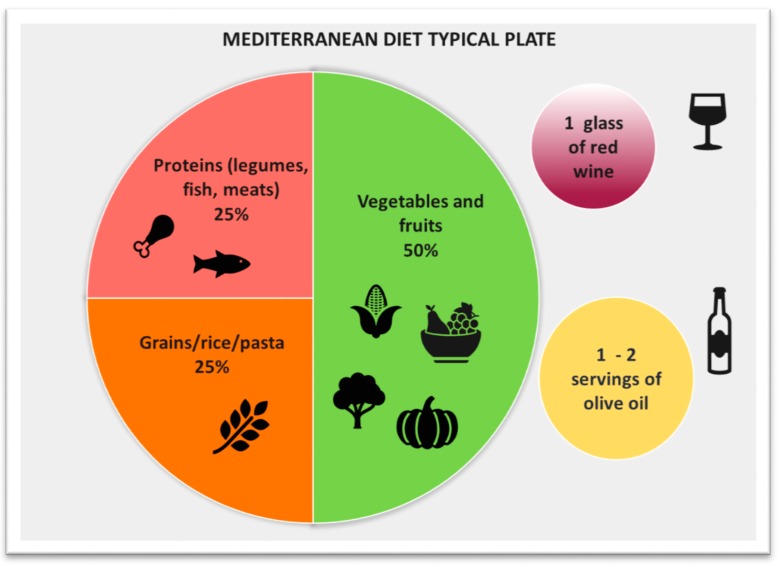
Typical composition of a meal in a Mediterranean dietary pattern.

The MD is defined as “a plant-based diet characterized by a high ratio of mono-unsaturated fatty acids (MUFA) to saturated fatty acids (SFA) with a total fat accounting for 30%–40% of daily energy consumption” (Figure 1). The MD is rich in olive oil, which is the main source of added fat together with nuts, has a high percentage of fibers, mainly obtained from vegetables, whole grains and legumes, and is rich in fish and seafood, while meat and dairy products are consumed in a lower percentage. It is therefore a high-fat diet, with fat comprising 35%–45% of the total energy intake, at least half of which should be from MUFAs. Carbohydrates constitute 35%–40% and protein 15%–20% of the energy intake. As far as alcohol use is concerned, a moderate intake of ethanol is an additional characteristic of the MD (in particular, wine) [24]. A synthetic example of the recommended composition of a meal in the MD is given in Figure 2.

The MD is therefore rich in macronutrients that have been shown to have a beneficial effect on glucidic and lipidic metabolism and, consequently, on fatty liver disease [13]. In particular, the MD is high in mono-unsaturated fatty acids (MUFAs), poly-unsaturated fatty acids (PUFAs), and fibers, and low in refined sugars and added fructose. MUFAs reduce risk factors for MetS which are linked to NAFLD, such as waist circumference, high-density lipoprotein cholesterol, triglycerides, and glucose, and therefore have a protective effect against cardiovascular events [13]. PUFAs, particularly omega-3 fatty acids, have been shown to be beneficial in preventing cardiovascular events via an improvement of insulin-sensitivity, an anti-inflammatory effect, and a reduction in oxidative stress [25,26,27]. Dietary fibers reduce the risk of MetS components (type 2 diabetes, dyslipidemia) and cardio-vascular risk, and may be involved in a reduction of the risk for NAFLD via a modulation of gut microbiota, thus leading to the increased production of short-chain-fatty acids and phenolic compounds, which have antioxidant properties [28]. These effects are important goals in NAFLD patients since cardiovascular events are the major complication leading to death [29]. Furthermore, it has been shown that hepatic steatosis is reduced in patients consuming high amounts of MUFAs [30]. In particular, in a cross-over study including 12 patients, Ryan et al. found that a MD pattern rich in MUFAs, mainly from olive oil, was associated with a reduction in steatosis (analyzed by magnetic resonance) even if a reduction in body weight was not achieved [31]. This study had the limitation of including a low number of patients but its results were strengthened by the randomized cross-over design and the use of magnetic resonance spectroscopy for the measurement of hepatic steatosis. It needs to be emphasized that the protective effect of this MD pattern may be driven not only by MUFAs, but also by the phenolic compounds in which olive oil is rich, exerting anti-inflammatory, metabolic, and antioxidant effects [32,33,34,35].

PUFAs, and in particular omega-3 fatty acids, exert a similar effect on NAFLD. More precisely, a diet rich in omega-3-fatty acids has been shown to be protective against fatty liver as it prevents hepatic fat accumulation and reduces hepatic steatosis [36,37,38,39,40]. Additionally, it seems to be important that a low omega-6/omega-3 ratio is maintained in order to obtain benefit from omega-3 PUFAs. This evidence comes from observational studies showing higher rates of NAFLD and NASH in patients following a diet with a higher omega-6/omega-3 ratio [41,42,43]. However, no benefits have been described regarding the regression of fibrosis nor the regression of NASH [40,44].

An additional benefit of the MD on fatty liver disease is related to its low content of refined sugars, fructose, and high content of complex carbohydrates and fibers. Epidemiologic reports have identified a directly proportional association between refined sugar, particularly high-fructose corn syrup, and the risk for NAFLD, even in the absence of additional risk factors [45,46,47,48]. Moreover, Western dietary patterns, which are typically rich in added fructose and corn syrup, and added fructose-rich diets have been proven to increase hepatic steatosis in humans and in mice models, whereas a negative correlation was found between low-carbohydrate diets and the risk for hepatic steatosis [49,50,51]. Additionally, it has been widely demonstrated that processed foods, especially soft-drinks, promote liver fat accumulation through de-novo lipogenesis of fructose in the liver, a mechanism that is already altered in NAFLD patients [52,53,54].

Unlike the Western diet, the MD is rich in whole grains, which have a high content of fibers. Whole grains and fibers may be beneficial in NAFLD patients for different reasons. The first is that they have less energy density and induce more satiety than refined carbohydrates. Secondly, they have prebiotic effects that modulate gut microbiota, which may play a role in the pathogenesis of NAFLD and its progression. Finally, they have well known protective effects against the risk for diabetes, dyslipidemia, cardio-vascular diseases, and all-cause mortality [55,56]. Animal models have shown that prebiotic fibers may modulate the human microbiome, leading to a proliferation of bacterial strains that produce short-chain fatty acids with anti-inflammatory properties, and whose metabolism leads to reduced serum cholesterol levels and triglycerides in the liver [28,57,58,59].

A minimal amount of meat and processed meat intake is another characteristic of the MD. Meat contains cholesterol, saturated fatty acids (SFAs) which are relevant in NAFLD and some case-control and cross-sectional studies have shown the presence of a correlation between meat consumption and NAFLD [41,60].

The MD consents a moderate amount of alcoholic units per day. Although there is a lack of studies with convincing evidence to support the benefits versus harms of ethanol intake, light alcohol intake seems safe in NAFLD non cirrhotic patients [61,62]. Indeed, a recent retrospective study on the effects of alcohol consumption on survival in NAFLD-patients, reported that drinking 0.5–1.5 drinks per day decreases the risk of overall mortality by 41% (hazard ratio (HR) = 0.59, 95% confidence interval (CI) 0.40–0.85, *p* = 0.005) compared with not drinking. On the contrary, drinking ≥1.5 drinks per day showed a harmful effect [63]. However, this study focused on the effects of alcohol on overall survival and its effects on liver disease progression are not described.

Lastly, there is no specific recommendation about coffee consumption in a typical Mediterranean dietary pattern. However, coffee may have beneficial effects on NAFLD prevention and NASH severity [64,65,66]. Interestingly, Molloy et al. showed that coffee consumption was significantly associated with a reduced prevalence of steatosis and a lower severity NASH. More specifically, when comparing patients with bland steatosis/not-NASH to those with NASH stage 0–1 and also when comparing patients with NASH stage 0–1 to those with NASH stage 2–4, there was a significant difference in coffee consumption between the two groups (*p* = 0.005 and *p* = 0.016, respectively). Importantly, this study also reported a significant correlation between coffee consumption and hepatic fibrosis (r = −0.215; *p* = 0.035) [66]. Notably, an inverse relationship between coffee and HCC risk has been shown [67,68,69]. Indeed a recent metanalysis including twelve studies on HCC (3,414 patients) and six studies on chronic liver disease (1,463 patients) evidenced that the summary relative risks for HCC were 0.66 (95% confidence interval (CI): 0.55–0.78) for regular, 0.78 (95% CI: 0.66–0.91) for low, and 0.50 (95% CI: 0.43–0.58) for high coffee consumption, respectively [70]. Therefore, considering also that evidence supports the cardioprotective effects of coffee and a reduced prevalence of MetS in coffee-consumers [71,72,73], a moderate coffee intake (2–3 cups/day) can safely be recommended.

## 3. Current Evidence about the Effects of the Mediterranean Diet on NAFLD

On the basis of the reported benefits of the MD components on the features of the MetS, including NAFLD, the MD has been proposed as the diet of choice for the dietary treatment of hepatic steatosis [74]. Herein, we report the current evidence on the liver-related outcomes of the MD diet. The evidence for the protective effects in terms of cardiovascular events are not the aim of this review and have been extensively reported elsewhere [75]. Indeed, the indication for the MD as a treatment for NAFLD is recent and is based on few observational and interventional studies, of which only two included the performance of a liver biopsy. Most of the studies have focused on assessing the role of MD, and variants of it, on the prevention and reduction of steatosis, as shown in Table 1.

In a case-control study, a Mediterranean dietary and lifestyle pattern was associated to a lower likelihood of having steatosis, independently from body weight and energy intake [85]. The investigators included 100 ultrasound-proven NAFLD patients and 55 healthy controls; dietary habits were assessed through a semi-quantitative food questionnaire. Habitual total night sleep hours as well as naps were reported as well. A significant association was found between a dietary pattern with the characteristics of a MD with a higher consumption of sweets, lower consumption of vegetables and nuts (all *p* < 0.005), and lower physical activity (*p* = 0.006) and NAFLD. Likewise, sub-optimal sleep duration was also associated with NAFLD (*p* = 0.005, 95% CI 0.14–1.01).

In a prospective study, a “Spanish Ketogenic Mediterranean Diet”, rich in omega-3 fish oil and olive oil, low in carbohydrates and meat, was prescribed as a treatment in 14 obese men with MetS and NAFLD (diagnosed by ultrasound and ALT levels >40 U/L). Steatosis degree (measured by means of ultrasound scanning using a 4-point validated scale [86]) was significantly reduced in the treatment group (*p* < 0.001), and complete steatosis remission was achieved in 21.4% of patients with a 92.86% overall reduction; accordingly, a significant reduction of hepatic necrosis enzymes was reported (ALT from 71 to 39 U/L, AST from 47 to 29 U/L; *p* < 0.001) [76]. Similar results were obtained by Ryan et al. in a pilot cross-over study including 12 obese non-diabetic men with biopsy-proven NAFLD [31]. In this case, all patients undertook a MD and a control diet (low-fat/high carbohydrate diet based on the American Heart Association Diet and Australian National Heart Foundation) in random order for 6 weeks, with a 6 week wash-out period in between. Hepatic fat content was measured by means of magnetic resonance H-spectroscopy, which calculates intrahepatic lipid that is considered the gold standard for quantification of hepatic steatosis. This parameter was significantly improved after the MD treatment period (mean i intrahepatic lipid decreased by approximately 39% from baseline, *p* < 0.05) as well as insulin-resistance (*p* < 0.001), even in the absence of weigh reduction. The control diet, surprisingly, failed to achieve such endpoints, and as far as liver enzymes are concerned, no reduction was observed after either dietary intervention; however, this latter finding might be due to the already normal levels of the liver enzymes at enrollment. Another recent randomized controlled trial investigating the effects of the MD diet and a low-fat diet on fatty liver and cardiometabolic risk factors, proved the effectiveness of MD in the reduction of hepatic fat, measured by means of magnetic resonance spectroscopy. Forty-nine subjects with magnetic resonance-proven NAFLD were randomized to a 12-week blinded dietary intervention (MD versus low-fat): at week 12, hepatic steatosis and liver enzymes were significantly reduced in both groups (*p* < 0.001) and there was no difference in liver fat reduction between the two interventions, with an overall mean reduction of 25.0% [12]. Interestingly, these results were achieved even in the absence of significant weight loss as in this cohort the average weight loss was around 2% from the baseline, which is not the weight loss target (5–7%) recommended by the latest EASL-guidelines.

Additional evidence for the benefit of the MD in fatty liver disease has been reported in a recent randomized clinical trial which was conducted on 50 overweight patients whose liver fat was assessed with ultrasonography and staged by the Hamaguchi score (a highly specific and sensitive scale which uses a 6-point scoring system based on hepato-renal echo contrast, liver brightness, deep attenuation and vascular blurring) [77]. Patients were randomized to three groups (MD alone/MD and antioxidant supplementation with milk thistle/control) and followed a 6-month MD with/without an antioxidant supplement. After 6 months of intervention, a significantly (*p* = 0.0001) decreased hepatic fat content, assessed by ultrasonography and as compared to baseline, was reported for patients on MD but not for controls. Likely, the fatty liver index (FLI)-which is a validated algorithm (range, 0–100) based on body mass index, waist circumference, triglycerides and gamma-glutamyl-transpeptidase values-decreased significantly [77,87].

The MD pattern is not only characterized by diet but also by regular physical activity so that it can be considered a lifestyle and not a simple dietary pattern. In accordance with this concept, some authors have reported that the beneficial effects of the MD may be mediated more intensely by the MD lifestyle rather than by the diet itself [34,80,88]. Indeed, Abenavoli et al. described a case series including 7 obese patients who were prescribed a strict MD regimen, physical activity and antioxidant supplement (milk thistle): at 6-week evaluation all patients had improved Hamaguchi score, as it ranged from 1–3 at baseline and 0–1 at the end of follow up [89].

Additional evidence for an amelioration of steatosis in patients on a MD lifestyle is provided by the results of a study by Gelli et al. who analyzed the clinical effectiveness of nutritional counseling on reduction of NAFLD severity in 46 adults diagnosed with steatosis by ultrasonography [79]. In this non-controlled study, the percentage of patients with severe steatosis decreased from 52% to 9%, with a remission of steatosis in 20% of the study cohort. More in detail, the fatty liver index decreased from a mean value of 61.55 ± 26.76 to 48.83 ± 31.01 (*p* < 0.01). Regarding liver enzymes, aspartate and alanine aminotransferase, and gamma-glutamyl-transpeptidase significantly decreased during treatment but the normalization was particularly evident for alanine aminotransferase whose prevalence of alteration decreased from 67% at baseline to 11% following treatment; in particular, aspartate aminotransferase mean values decreased from 36 ± 21 at baseline to 25 ± 11 at 6 months (*p* < 0.01), while alanine aminotransferase values decreased from 66±31 to 37 ± 19 at 6 months (*p* < 0.01) and gamma-glutamyl-transpeptidase decreased from 60 ± 61 to 40 ± 35 (*p* < 0.01). The results of the study also highlighted the benefit of a nutritional counseling which increased the adherence to the dietetic and lifestyle regimen.

Similarly to Gelli et al. also Trovato et al. reported that an intervention focused to increase the adherence to the MD [assessed by the Adherence to Mediterranean Diet Score (AMDS)] and the level of physical exercise significantly decreased hepatic steatosis, measured by Bright Liver Score (BLS) after 6 months in 90 non-diabetic obese patients (BMI 31.0 ± 5.2) [78]. Interestingly, at a multiple linear-regression age-balanced analysis, the changes in AMDS were related to the reduction in hepatic fat content independently of physical activity. However, a potential limitation of this study is the fact that the BLS is not accurate in the staging of fatty liver and is indeed not included among the validated steatosis scores by the most recent EASL/EASD/EASO guidelines [74].

Additional evidence about the effect of a variant of the MD, i.e., a low-glycemic MD, on NAFLD severity, measured by means of ultrasonography was reported by an Italian randomized controlled trial including 98 men and women with moderate or severe steatosis [81]. Low-glycemic MD, not energy-restricted, was effective in the reduction of FLI and alanine aminotransferase levels. More specifically, FLI median and interquantile range significantly decreased from baseline values of 82.28 (70.31–90.38) to 6-month values of 57.72 (27.33–73.14, *p* < 0.05), while alanine aminotransferase levels decreased from 48 U/L to 39 U/L (*p* < 0.05) [83].

Interestingly, a recent randomized controlled trial addressed the question whether a reduction in hepatic fat content (HFC) has prognostic significance beyond the loss of visceral adipose tissue [11]. In this study, 278 participants with abdominal obesity or dyslipidemia were randomized to low-fat or Mediterranean/low-carbohydrate (MD/LC + 28 g walnuts/day) diets with or without moderate physical activity. Hepatic fat content and abdominal fat-depots were measured using magnetic resonance imaging. After a 6- and 18-month follow-up period, while the reduction in hepatic fat content was similar between physical activity groups, MED/LC induced a greater proportion of decrease in hepatic fat content (*p* = 0.036), and a greater improvement in cardiometabolic risk parameters (*p* < 0.05) than the low-fat diet, even after controlling for visceral adipose tissue changes. Importantly, after controlling for visceral adipose tissue loss, decreased hepatic fat content also remained independently associated with reductions in liver enzymes and glycated hemoglobin. These findings suggest that the beneficial effects of the MD/LC diet on specific cardiometabolic parameters may be mediated more by decreases in hepatic fat content than visceral adipose tissue loss.

Additional evidence about the effects of the MD on fatty liver disease in the mid- and long-term (12 months) will be shown in the near future by the results of the MEDINA study, a randomized controlled trial including 94 patients with NAFLD and insulin-resistance who have been randomized into two groups: MD or low-fat diet [84]. This trial aims to demonstrate, in a large cohort of participants with NALFD, that the MD can result in significant benefits in liver fat and insulin sensitivity and that these changes are sustained at 12 months, independently of weight loss [90].

As the reduction in liver enzymes is concerned, more evidence comes from a recent metanalysis including twenty randomized controlled trials including an overall 1073 NAFLD patients which showed that, compared to standard care—i.e., general advice for healthy diet and lifestyle—a more scheduled exercise and dietetic program improved aminotransferases, hepatic fat, and NAFLD activity score, irrespectively of weight change [81]. Regarding the type of exercise, aerobic compared to resistance exercise did not yield any superior improvements on liver parameters, whereas moderate-to-high volume moderate-intensity continuous training was more beneficial compared to continuous low-to-moderate-volume moderate-intensity training or high intensity interval training [80]. The same group conducted a randomized, controlled, single-blind clinical trial investigating the effect of the MD or of Mediterranean lifestyle in NAFLD patients. Sixty-three overweight or obese patients with ultrasonography-proven NAFLD and elevated alanine aminotransferase and/or gamma-glutamlyl-transpeptidase were randomized to a control group, or to MD or Mediterranean lifestyle (ML) groups. Participants included in the MD and ML arms participated in group sessions aiming at weight loss and increasing adherence to the MD. Additionally the ML was given guidance for increasing physical activity and improving sleep habits, while patients in the control group received only general written information for a healthy lifestyle. At the end of the six-month follow-up period, the ML group showed significant improvements in alanine aminotransferase levels and liver stiffness compared with the control group, while in contrast the MD group failed to achieve these endpoints. Indeed, the ML group achieved significant reduction in alanine aminotransferase levels (*p* = 0.009) and liver stiffness (*p* = 0.004) as compared to the control group (after adjusting for percentage of weight loss and baseline values) whereas the MD group improved only liver stiffness compared with the control group (*p* < 0.001). No differences were noticed regarding the study’s primary and secondary outcomes (clinically significant decrease in alanine aminotransferase levels and significant improvements in liver stiffness, NAFLD fibrosis score and gamma-glutamlyl-transpeptidase values) between the MD and ML groups, except that after controlling for baseline values and the proportion of weight loss-patients, the ML group tended to have a higher likelihood of halving their ALT levels compared with the MD group, though not significantly so (OR = 0.27; 95% CI, 0.07–1.03). This evidence suggests that a lifestyle-guided intervention may be the optimal treatment strategy for NAFLD [82].

To the best of our knowledge, the effects of the MD on NASH have been investigated only by two studies [91,92]. Kontogianni et al. conducted an observational study which showed that a higher adherence to the MD was associated with lesser degree of insulin resistance, as measured by the HOMA-score (*p* = 0.005), and less severe steatosis at histology (*p* = 0.08) among patients with NAFLD [91]. Moreover, a lower adherence to the MD was associated with a higher risk of progression towards NASH. More in detail, logistic regression analysis revealed that one unit increase in the MedDietScore (a score that assesses the adherence to MD via a questionnaire) was associated with 36% lower likelihood of having NASH, after adjusting for sex and abdominal fat (OR: 0.64; 95% CI, 0.45–0.92, *p* = 0.02) [83]. Similar results were reported by Della Corte et al. in a cohort of 243 obese pediatric patients [92]. The level of adherence to the MD was evaluated with a questionnaire, the Mediterranean Diet Quality Index for children and adolescents (KIDMED). A low KIDMED was significantly higher in patients with NASH compared to patients without NASH, with fatty liver and without fatty liver (100% versus 29.79% versus 37.88% versus 9.10%, *p* = 0.01) and correlated with NAFLD activity score >5 and to grade 2 fibrosis [84].

The only study that has described the effects on liver stiffness so far is a prospective study by Abenavoli et al. aiming at the comparison between the effects of the MD with or without an antioxidant complex supplement in overweight patients suffering from NAFLD [89]. The study proved that the MD alone or in association with the antioxidant complex, improved lipid profile, insulin resistance and reduced hepatic fat accumulation and liver stiffness (reductions in transient elastography of approximately 21%–17% in the MD groups were shown whereas the control group showed an increase in liver stiffness by 8.7%, *p* = 0.0001) [77].

EASL/EASD/EASO NAFLD guidelines recommend diet and physical activity as the best treatment for steatosis. However, a specific training program is not described. Studies investigating the effects of exercise on NAFLD have been conducted with similar results suggesting that both aerobic and resistance training reduce hepatic steatosis [91,92,93,94,95]. Recent metanalysis confirmed this evidence, reporting that independently from which type of exercise program is followed (aerobic vs. resistance) the steatosis grade is significantly reduced if a scheme including at least 3 weekly sessions of 40–45 min for 12weeks is followed [96,97]. Apparently, males tend to have a greater benefit in terms of hepatic fat reduction from aerobic exercise, whereas women have the best benefit from resistance training [96]. Obviously, high compliance to exercise is needed, and a significant proportion of NAFLD patients may have difficulties following aerobic exercise training because of concomitant cardiovascular comorbidities or physical disability due to obesity. Therefore, when considering which training is the best, an individualized decision has to be made, taking into account patients’ comorbidities. Lastly, the principal end-point of all the studies assessed reported positive outcomes (i.e., decrease in aminotransferase, improvement in steatosis or histology) in patients who experienced weight loss, and all the studies combined diet and exercise.

## 4. Future Research

NAFLD is going to become a major global health concern, and thus there is an increasing need to raise the awareness about the prevention and treatment of this disease, which nowadays seems to be very low [98].

Although weight loss can be an effective treatment, for most patients it is a difficult goal to achieve and maintain. MD has recently been suggested as the diet of choice for NAFLD treatment but the evidence behind this recommendation is very low as most of the studies that have addressed this issue included few patients, differed in the inclusion/exclusion criteria, in the methods by which steatosis was measured, and in the measurement of outcomes.

In order to have approved regimens for the treatment of NASH/NAFLD, therefore, more studies with large cohorts of patients, with consistent clinical endpoints as well as inclusion criteria and methods, are needed. Currently, the resolution of NASH and/or reduction of fibrosis stage are the accepted endpoints by the regulatory authorities since NASH progression and fibrosis stage have prognostic implications. In this context, although histologic NASH has been associated with advanced fibrosis, it is not an independent predictor of long-term mortality. In contrast, there are significant data to suggest that the fibrosis stage is the only robust and independent predictor of liver-related mortality, and it is auspicated that in the future, adequately powered studies will disclose whether the MD may truly represent a viable therapeutic means able to obtain these relevant goals [99,100].

## Figures and Tables

**Table 1:** Recent studies about the effects of the Mediterranean diet on liver-related outcomes in NAFLD and NASH.

Reference	Design	Dietary Composition	Study Aim	*N* Patients, Age (Median)	NAFLD Diagnosis and Assessment at Follow Up	Baseline Parameters	Main Results
Perez-Guisado et al. [76]	Prospective study, 12 weeks of dietary treatment. No physical activity Follow up at 12 weeks	Unlimited calories. High doses of virgin olive oil (min 30 mL/day) and ω-3 fatty acids from fish as main source of fat, fish as the main source of protein, green vegetables and salads as the main source of carbohydrates (max. 30 g/day)	Assessment of Spanish ketogenic MD effects on steatosis degree, measured by US and serologic measures of liver function	14 41 years	NAFLD was diagnosed by ALT levels >40U/L and steatosis on US. Steatosis degree was assessed by US using a four-point validated scale	BMI 36.5 ± 0.54 kg/m^2^ ALT 71.9 ± 3.5 U/L AST 47.7 ± 2.8 U/L Not stated whether patients had diabetes	-Complete fatty liver regression (21.4% of the patients) with an overall reduction in 92.86% of patients (*p* < 0.001) -Significant (*p*<0.001) BM, AST and ALT reduction (mean BMI 32.4, AST 29 and ALT 37.0)
Ryan et al. [31]	Randomised cross-over trial: patients received six weeks of MD and six weeks of low fat/high carbohydrate diet. No physical activity Follow up at end of second diet	Unlimited calories. High in MUFAs from olive oil and ω-3 fatty acids from fish; approximate macronutrient composition 40% fat, 40%carbs, 20% proteins	Assessment of MD on steatosis degree, measured by MRI, and on insulin sensitivity	12 55 years	Biopsy-proven NAFLD diagnosis Steatosis degree was assessed by MRI	BMI 32.0 ± 4.0 kg/m^2^ ALT 49.0 ± 23 U/L No patients were diabetic (No significant differences between groups)	−39% relative reduction in steatosis in MD intervention group (*p* < 0.05) -No significant reduction in BMI/ALT in either diet -Improved insulin sensitivity in MD (*p* = 0.03)
Abenavoli et al. [77]	Randomised clinical trial. Physical activity recommended Low-calorie MD was prescribed to Group A and B patients for 6 months. Group B received also antioxidant complex. Group C received no treatment/advice. Follow up at 6 months	Low calorie MD with carbohydrates (50%–60%), proteins (15%–20%, about 50% of which were vegetable proteins), MUFAs and PUFAs (less than 30%), saturated fat (less than 10%), cholesterol (less than 300 mg/day) and fibers (25–30 g/day. Treatment prescribed for six months.	Evaluate the effects of antioxidant complex associated with MD on liver fat accumulation, BMI, glucose, and lipid metabolism	50 Group A: 52 years Group B: 46 years Group C: 33 years	NAFLD was diagnosed by US Steatosis degree was assessed by FLI and US using the Hamaguchi score	BMI 31 (29–33) kg/m^2^ in group A; 29 (28–32) kg/m^2^ in group B; 29 (27–31) kg/m^2^ in group C AST and ALT median levels within normal range in all groups Not stated if patients were diabetic	Significant reductions in BMI (*p* = 0.0001), lipid profile (*p* < 0.001) and in FLI (*p* < 0.01) and US-Hamaguchi score (*p* = 0.0001) were reported in groups A and B but not in the control group.
Trovato et al. [78]	Prospective, observational No dietary treatment but behavoural and dietary counseling Follow up at six months	Behavioral and dietary counseling	To evaluate the effectiveness of an intervention focused to increase the Adherence to Mediterranean Diet Score (AMDS) and the level of physical exercise	90 50 years	NAFLD was diagnosed by US Steatosis degree was assessed by BLS	BMI 31 ± 5 kg/m^2^ ALT 23.3 U/L BLS 1.96 ± 0.69 No patient had diabetes	Significant reduction in BMI (*p* < 0.0001) and BLS (*p* < 0.0001). No significant reductions in ALT Significant reductions in liver stiffness, assed by TE
Gelli et al. [79]	Prospective, observational Restricted calories MD for six months Follow up at six months	Calorie restriction: maximum calorie reduction, 500 kcal/day. MD: 55%–60% of carbohydrates of which 80% complex carbohydrates (bread, pasta, rice), 10%–15% of proteins about 60% of animal origin (especially white meat, fish), 25%–30% fat (mostly olive oil)	End-points: (1) reduction of at least 1 unit of steatosis grade; (2) A 7% weight reduction; (3) normalization or improvement of metabolic indexes and (4) normalization or improvement of ALT, AST, GGT.	46 47 years	NAFLD was diagnosed by US Steatosis degree was assessed by a four-grade US scale	BMI 29.3 kg/m^2^ ALT was abnormal in 67% patients whereas AST and GGT were abnormal in 26% and 34% of patients, respectively 8% of patients had diabetes	-Steatosis regression 20% -In more than 80% of patients at least one grade reduction of steatosis degree -BMI decreased on average from 29.3 ± 6.11 to 27.5 ± 6.21 (*p* < 0.01) -Significant reduction of ALT, AST and GGT (*p* < 0.01)
Katsagoni et al. [80]	Metanalysis of RCTs about lifestyle interventions in NAFLD		RCTs of intervention with exercise and/or diet in NAFLD-patients, in English language were included. Aim: evaluate the efficacy of lifestyle interventions on liver, anthropometric and metabolic parameters.	20 RCT with 1073 patients were included	NAFLD diagnosis by means of US or MRI or biopsy		Combination of exercise and diet decreased ALT levels (*p* < 0.01) and improved NAFLD activity score (SMD = −0.61, 95% CI: −1.09to −0.13). Moderate-carbohydrate diets yielded similar changes in liver enzymes compared to low/moderate-fat diets
Misciagna et al. [81]	Double blind RCT LGIMD or INRAN diet for six months Follow up at six months	Energy restriction. Low glycemic Index MD vs. Italian National Research Institute for Foods and Nutrition (INRAN) diet.	To estimate the effect of a LGIMD on steatosis as measured by US	98 Median age not available	NAFLD diagnosis by US Steatosis degree with an US based scale	4% of patients had normal BMI values, 26% were overweight and 70% were obese. None was diabetic. All patients had moderate/severe steatosis on US.	FLI and steatosis degree significantly decreased in both diets (*p* < 0.05) but the decrease was greater in the LGIMD group
Katsagoni et al. [82]	Single blind RCT Patients randomized in control group, MD group and Mediterranean lifestyle (ML) group. MD and ML group also received regular counseling during follow up, including training and sleep duration counseling for the latter Follow up at six months	All groups received energy restriction and similar dietetic regimen with 45% carbs, 20% protein and 35% lipids MD and ML groups were also counseled regularly	To estimate the effect of MD or Mediterranean lifestyle in NAFLD patients. vStudy primary outcomes (i.e., ALT<40U/l and 50% reduction of ALT levels)	63 50 years	NAFLD diagnosis based on the following criteria: elevated alanine aminotransferase (ALT) and/or γ-glutamyl-transpeptidase (GGT) levels, evidence of hepatic steatosis on ultrasound and/or compatible liver histology	BMI 31.8 ± 4.5 kg/m^2^ Not stated if patients were diabetic	Greater BMI reductions (*p* = 0·008) in MD and ML compared with control ML showed significant improvements compared with the control (*p* = 0.009), and a higher tendency compared to MD (OR = 0·27; 95% CI 0·07, 1·03, in ALT reduction. Both MD and ML improved liver stiffness in TE
Properzi et al. [12]	Single blind RCT Randomization to MD vs. Low-fat diet (LFD )Follow up at 12 weeks	LFD composition: 50% carbs, 30% fat (with <10% of energy as saturated fat), and 20% protein MD composition: 40% carbs, 35–40% fat (with <10% of energy as saturated fat), and 20% protein	Investigate the effect of two ad libitum isocaloric diets (MD or LFD) on hepatic steatosis	48 52 years	NAFLD diagnosis was made by MRI	BMI 30.2 kg/m^2^ in LFD BMI 31.5 kg/m^2^ in MD ALT 66.8 U/L in LFD ALT 76.5 U/L in MD 28% patients in LFD and 30% in MD had diabetes	Hepatic fat content reduced significantly in both groups (*p* < 0.01), with no difference between groups (*P* = 0.32). Liver enzymes improved significantly in both groups. Weight loss: minimal and not different between groups
Gepner et al. [11]	Single blind RCT Randomization to M-low carbs (MD/LC) vs. LFD. After six months each diet group was further randomized into added physical activity or no added PA groups for another 12 months Follow up at 18 months	-LF diet: fat maximum 30% of calories, with up to 10% of saturated fat, no more than 300 mg/day of cholesterol, increased dietary fiber.-MD/LC diet: MD with less than 70 g/day of carbs, increased protein and fat intake plus 28 g of walnuts/day	Investigate if hepatic fat loss in response to dietary interventions induces specific beneficial effects independently of visceral abdominal fat (VAT) changes.	278 48 years	NAFLD diagnosis was made by MRI	BMI 30.8 ± 3.8 kg/m^2^ Overall, 53% of patients had MRI-proven steatosis. All patients had abdominal obesity. 11% of patients had diabetes	Hepatic fat substantially decreased after 18 months (4.0% absolute units (29% Relatively); *p* < 0.001 vs. baseline) in entire cohort (also in patients without steatosis, i.e., HF < 5%). Hepatic fat was substantially reduced by diet-induced moderate weight loss and more effectively by the MED/LC diet than the LF diet (*p* = 0.036), independently of VAT changes.
Kontogianni et al. [83]	Retrospective study Adherence to MD and physical activity over the last 12 months were assessed		To investigate the associations between adherence to the MD(estimated with MedDietScore) and histological characteristics of NAFLD	73 45 years	NAFLD diagnosis based on the following criteria: elevated alanine aminotransferase (ALT) and/or γ-glutamyl-transpeptidase (GGT) levels, evidence of hepatic steatosis on ultrasound and/or compatible liver histology	BMI 29.7 ± 4.6 kg/m^2^ No patient had diabetes. 58 patients were matched with 58 healthy controls for age, sex and BMI. 63% of patient had NASH	MedDietScore negatively correlated to ALT (*p* = 0.03) and severity of steatosis (*p* = 0.006) Patients with NASH exhibited lower adherence to MD (29.3 +/− 3.2 vs. 34.1 +/− 4.4, p = 0.004) compared to those with simple fatty liver and had higher BMI (*p* = 0.028). No difference in the MedDietScore was observed between patients and controls
Della Corte et al. [84]	Observational study Adherence to MD was assessed at time of inclusion		To analyze the association between adherence to the MD(using the KIDMED Index) and NAFLD, with laboratory and histologic evaluation, in a group of children and adolescents with obesity.	243 Age: 10–17 years	NAFLD diagnosis was made on an US basis with/without abnormal transaminases	BMI 28.1 ± 6.3 kg/m^2^ No patient had diabetes 166 patients had steatosis, among them 53 had NASH	Low KIDMED score was significantly higher in patients with NASH (*p* = 0.001) and correlated with a NAFLD activity score (NAS) >5 (*p* = 0.001), and grade 2 fibrosis (*p* = 0.02). Low KIDMED correlates with fibrosis (OR 2.58, 95%CI 1.36–3.9, *p* = 0.05) and NAS (OR 1.03, 95%CI 0.9–2, *p* = 0.05)

MD, Mediterranean Diet; BMI, Body Mass Index; US, ultrasound scan; MRI, magnetic resonance imaging; ALT, alanine aminotransferase; AST, aspartate aminotransferase; GGT gammaglutamiltranspeptidase; NAFLD, nonalcoholic fatty liver disease; NASH, nonalcoholic steatohepatitis; MUFAs, monounsaturated fatty acids; FLI, fatty liver index; RCT, randomised controlled trial; TE, transient elastography.
